# *FANCA* Deficiency Induces Oncogenic R-Loop Dependent Synthetic Lethality with PARP1 Inhibitors

**DOI:** 10.21203/rs.3.rs-6080272/v1

**Published:** 2025-07-03

**Authors:** Gaorav Gupta, Qinhong Wang, Simon Ellington, Paolo Guerra, Faeze Gharibpoor, Dennis Simpson, Min-Guk Cho, Adriana Beltran

**Affiliations:** University of North Carolina at Chapel Hill; University of North Carolina at Chapel Hill; University of North Carolina at Chapel Hill; University of North Carolina at Chapel Hill; University of North Carolina at Chapel Hill; FCCC; University of North Carolina at Chapel Hill; University of North Carolina at Chapel Hill

## Abstract

Synthetic lethality (SL) underlies the success of PARP1 inhibitors (PARPi) in treating homologous recombination (HR) deficient cancers, but extending this paradigm to other DNA damage response (DDR) deficiencies has proven challenging. We performed an in vivo CRISPR screen to identify DDR gene mutations that both enhance tumorigenesis and confer sensitivity to PARPi. Our screen identified FANCA deficiency as a driver of PARPi SL that was validated across diverse human cancer models. FANCA deficiency does not impair HR but disrupts Okazaki fragment maturation (OFM), causing lagging strand gaps and RPA exhaustion upon PARPi treatment. These effects require FANCA interaction with FEN1, independently of its canonical role in interstrand crosslink repair. We find FANCA-mediated FEN1 recruitment is required for OFM at oncogene-associated R loops during PARPi treatment. These findings establish a novel and non-canonical function for FANCA in FEN1-mediated OFM that can be leveraged for PARPi synthetic lethality in FANCA-mutant cancers.

## Introduction

Synthetic lethality (SL) describes the phenomenon wherein the simultaneous disruption of two cellular pathways leads to loss of viability, whereas disruption of either pathway alone is tolerated^1–3^. This concept has been effectively applied to target *BRCA1/2*-deficient cancers using poly-ADP ribose polymerase 1 inhibitors (PARPi)^4–7^. Advances in RNA interference and CRISPR-based genetic screens have identified numerous additional gene mutations—including those not directly involved in homologous recombination (HR) DNA repair—that confer selective sensitivity to PARPi, as well as other DNA damaging therapeutics^8–10^. However, translating these SL findings from *in vitro* screens into meaningful clinical benefits has proven challenging.

The limited clinical applicability of SL-based approaches may stem from the inherent constraints of *in vitro* CRISPR screening methodologies. These screens are typically performed in established cancer cell line models over relatively short durations, with limited evaluation of how the induced mutations interact within the complex *in vivo* tumor ecosystem. In tumors, even modest growth disadvantages are subjected to strong negative selection, reducing the likelihood that disruptive mutations will persist. Consequently, clinically observed mutations in genes that scored highly in *in vitro* SL screens yet are also important for tumor fitness are more likely to represent non-disruptive passenger mutations that fail to induce SL with the drug of interest. Therefore, SL-based therapies can be anticipated to be most effective when directed against tumor suppressor or driver oncogenic mutations that confer a tumor growth advantage.

To address these limitations, we employed an *in vivo* CRISPR tumorigenesis screen targeting DNA damage response (DDR) genes in a murine breast cancer model driven by c-Myc overexpression and p53 deficiency^11,12^. By using this approach, we ensure that the mutant alleles being studied are compatible with, and often accelerate, tumor progression. We demonstrate the feasibility of discovering novel SL gene-therapy interactions by evaluating polyclonal tumors with CRISPR-induced DDR gene mutations treated with various DNA damage or repair-targeting therapies. Secondary validation screens and analysis of mutation prevalence in human solid tumors led us to nominate *FANCA* deficiency as a novel SL interaction with PARPi sensitivity.

The Fanconi Anemia (FA) pathway is a DNA repair pathway that is essential for DNA interstrand crosslink (ICL) repair, and genetic impairment of this pathway is associated with cancer predisposition^13–16^. The FA core complex—comprising FANCA, FANCB, FANCC, FANCE, FANCF, FANCG, FANCL, FANCM, FAAP20, FAAP100, and FAAP24—senses replication forks stalled at ICLs and monoubiquitylates the FANCI/FANCD2 (ID2) complex, which subsequently recruits downstream mediators of translesion synthesis (TLS) and HR repair^17–19^. Notably, FANCA forms a subcomplex with FANCG and FAAP20^17,20^ which is dispensable for the assembly and ubiquitin ligase activity of the FA core complex *in vitro,* raising the possibility of distinct molecular functions^21^. FANCA also plays roles in single-strand annealing during DSB repair^22,23^ and in augmenting FEN1 nuclease activity^24^, but whether these molecular functions regulate therapeutic sensitivity in tumors remains unknown.

Poly(ADP-ribose) polymerases (PARPs) are a superfamily of enzymes that modify proteins through mono- or poly(ADP-ribosyl)ation^25,26^. PARP1, a key member, binds to and is activated by various DNA substrates, including single-stranded DNA (ssDNA) gaps and nicks that accumulate during replication stress^27–29^. The synthetic lethality between *BRCA1/2* deficiency and PARPi was originally linked to the essential role of HR in repairing one-ended DSBs generated in PARPi-treated cells. While this DSB-focused model highlights DSBs as the primary source of PARPi-induced cytotoxicity, recent evidence suggests that ssDNA gaps also play a critical role in determining PARPi lethality. These ssDNA gaps can arise from defects in Okazaki fragment maturation (OFM) or reduced repair of ssDNA breaks^30–32^. Emerging data indicate that PARP1’s essential function in driving SL upon inhibition involves an alternative OFM pathway during lagging-strand DNA synthesis^27–29^. Inhibition of PARP1 in BRCA-deficient cells results in extensive daughter-strand gaps, triggering replication catastrophe, and ultimately SL^5,33,34^. Supporting this model, mutations that impair HR without disrupting lagging strand synthesis do not exhibit PARPi sensitivity^35–37^, whereas disruption of OFM pathways can sensitize cells to PARPi independently of HR deficiency^29,33,38^.

Here, we demonstrate that *FANCA* deficiency does not impair HR but disrupts OFM due to loss of FANCA-dependent FEN1 recruitment, which is dispensable for canonical ICL repair. FANCA-dependent FEN1 recruitment is critical for OFM when PARP1 is inhibited, particularly under replication stress driven by oncogene-induced transcriptional stress. Importantly, FANCA’s SL with PARPi is conditional on oncogenic stress and can be rescued by either RNase H overexpression or transient inhibition of RNA polymerase II-dependent transcription. Collectively, our findings reveal the crucial novel role for FANCA in FEN1-mediated lagging strand maturation during replication stress and position *FANCA* mutation as a targetable vulnerability to create SL with PARPi through the accumulation of ssDNA gaps.

## Results

### In vivo DDR-CRISPR screen identifies FANCA synthetic lethality with PARPi

**Figure 1:**
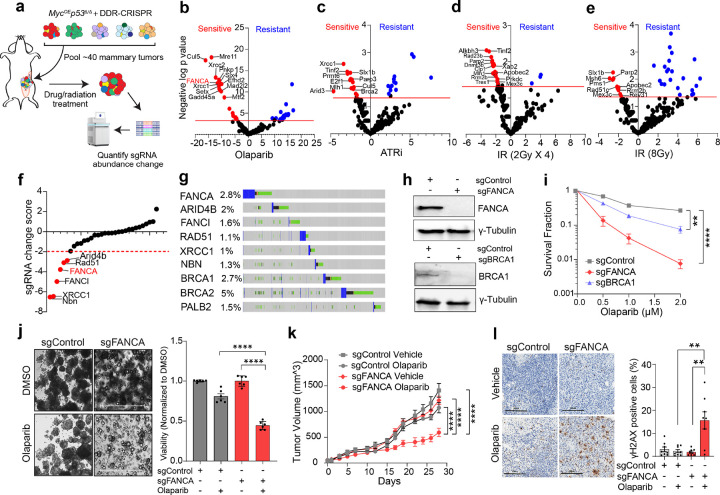
Synthetic lethality between *FANCA* deficiency and PARPi identified via *in vivo* and *in vitro* DDR-CRISPR screens. **a,** Schematic overview of the experimental setup for *in vivo* DDR-CRISPR screening. **b-e,** Volcano plots highlighting candidate DDR target genes based on negative log10 p-values from t-tests comparing DDR gene-targeting sgRNAs with non-targeting control sgRNAs. Treatments included PARPi (Olaparib), ATRi (AZD6738), and ionizing radiation (2Gy/day for four consecutive days or 8Gy in a single dose). Sensitizing sgRNAs were depleted whereas resistance-associated sgRNAs were enriched following therapy. **f,** *In vitro* secondary validation screening using a candidate DDR-CRISPR sgRNA sublibrary in two mouse cell lines (C8965L and C9266R) following Olaparib treatments identified candidate synthetic lethal gene targets using a negative 2-fold change cutoff. **g,** TCGA cohort analysis demonstrating *FANCA* as the most altered candidate gene in a pan-cancer cohort, comparable to *BRCA1, BRCA2,* and *PALB2*. **h,** Western blot analysis confirmed FANCA and BRCA1 knockout in MDA-MB-231 cells. **i,** Colony formation assays (CFAs) demonstrated reduced survival in *FANCA*-deficient MDA-MB 231 cells treated with PARPi (Olaparib: 0.5 μM, 1.0 μM, and 2.0 μM). Survival fractions were normalized to untreated controls. Data represent mean ± SEM (n=6 biological replicates); **p<0.01, ****p<0.0001 (unpaired two-tailed t-test). **j,** WHIM2 TNBC organoids with *FANCA* deficiency exhibited decreased viability after treatment with Olaparib (3μM, 7 days). Organoid viability was assessed using CellTiter-Glo (3D culture assay). Representative images and normalized viability data (mean ± SEM, n=6 biological replicates) are shown. Scale bars, 650 μm. **k,** *FANCA* deficiency sensitized xenograft tumors to PARPi. Tumor growth curves for mice treated with Olaparib (50mg/kg, oral gavage, daily for 28 days) are shown. Data represent mean ± SEM (n=6 tumors per group); ****p<0.0001 (unpaired two-tailed t-test). **l,** Immunohistochemistry (IHC) with DAB staining was performed to detect gH2AX in FFPE tumor tissue sections. Representative images and quantification of gH2AX-positive cells (mean ± SEM, n=8 tissue sections) are shown; **p<0.01 (unpaired two-tailed t-test). Scale bars, 200 μm.

We previously conducted an *in vivo* CRISPR screen to identify DDR gene mutations that promote breast tumorigenesis in transgenic mice with conditional alleles for Cas9, *c-Myc* overexpression, and *Trp53* deficiency (*Rosa26*^*LSL − Cas9/LSL−Myc*^*Trp53*^*fl/fl*^)^11,12^. 39 polyclonal DDR-mutant primary breast tumor lines established from this model were pooled and orthotopically implanted into the mammary glands of a cohort of female mice. Tumor-bearing mice were left untreated or treated with Olaparib (PARPi), ATR inhibitor (AZD6738), or ionizing radiation (2 Gy × 4 or 8 Gy × 1). Tumors were harvested upon reaching double their initial volume, typically 10–14 days after treatment initiation. Genomic DNA was isolated, and small guide RNA (sgRNA) abundance was quantified by high-throughput sequencing (Fig. 1a). Relative to untreated controls, therapy exposure led to significant depletion or enrichment of sgRNAs targeting specific DDR genes (Fig. 1b–e). PARPi treatment elicited the most pronounced changes in sgRNA abundance, underscoring the critical role of DDR gene mutations in determining PARPi sensitivity. Among negatively selected genes, 43 DDR genes—including well-characterized tumor suppressors and DDR regulators (e.g., *BRCA2, CUL5, MRE11, RNF4, XRCC2, FANCA, XRCC1, SLX4, RAD54B)* showed significant reductions in sgRNA abundance following PARPi treatment.

To further confirm the SL gene targets, we conducted a secondary validation screen using a mini-CRISPR library targeting 31 SL gene candidates from the PARPi-treated group that were also prioritized based on mutation prevalence in human cancers, as well as five control sgRNAs. This sgRNA library was transduced into two Myc^OE^p53^Δ/Δ^ mouse mammary tumor cell lines established from this model (C8965L and C9266R). After puromycin selection, cells were passaged for eight population doublings with or without Olaparib (0.5μM). Subsequent NGS analysis identified sgRNAs that were depleted following *in vitro* PARPi treatment (Fig. 1f). Importantly, among the validated SL gene targets, *FANCA* emerged as the most frequently disrupted gene in a pan-cancer cohort from The Cancer Genome Atlas, with a prevalence of 2.8% when including deep copy number loss, frameshift, splice-site, or missense mutations, comparable to *BRCA1* (2.7%) and *BRCA2* (5%) (Fig. 1g). Based on its secondary validation and high prevalence in human cancers, we prioritized *FANCA* deficiency for further investigation of PARPi synthetic lethality.

### FANCA deficiency induces PARPi synthetic lethality in human cancer models

To validate the synthetic lethality of *FANCA* deficiency with PARPi in human cancer models, we generated *FANCA*-knockout subclones of the triple-negative breast cancer (TNBC) cell lines MDA-MB-231 and BT-549 using CRISPR/Cas9. Single-cell clones were isolated and evaluated by Western blot analysis for *FANCA* knockout efficiency (Fig. 1h). Colony formation assays (CFAs) demonstrated that *FANCA* deficiency significantly sensitized both MDA-MB-231 and BT-549 cell lines to PARPi (Fig. 1i and Extended Data Fig. 1a-c). Cell-Titer Glo assays corroborated these findings, showing significantly reduced viability of *FANCA*-deficient MDA-MB-231 and BT-549 cells following eight days of Olaparib treatment (Extended Data Fig. 1d,e). Notably, while *BRCA1* knockout also sensitized MDA-MB-231 cells to Olaparib, the effect was less pronounced than with *FANCA* deficiency (Fig. 1h,i and Extended Data Fig. 1d).

To extend these findings in a more clinically relevant context, we utilized the WHIM2 patient-derived xenograft model of chemoresistant basal subtype TNBC, cultured as organoids^39,40^. Using CRISPR/Cas9, we generated *FANCA*-deficient isogenic WHIM2 organoid sublines, and PARPi treatment significantly impaired viability of established *FANCA*-deficient WHIM2 organoids and reduced organoid-initiating potential (Fig. 1j and Extended Data Fig. 1f,g). Flow cytometry revealed no significant differences in cell cycle distribution between *FANCA*-deficient and control cells (Extended Data Fig. 1h), indicating that *FANCA* deficiency enhances PARPi sensitivity independently of cell cycle alterations.

Having established the synthetic lethality of PARPi in isogenic *FANCA*-deficient clones *in vitro*, we next sought to verify these effects *in vivo*. Orthotopic implantation of *FANCA*-deficient MDA-MB-231 cells into *NOD/RAG1* female mice also demonstrated synthetic lethality with PARPi (Fig. 1k). Olaparib treatment, initiated when tumors reached 5 mm, significantly impaired the growth of *FANCA*-deficient tumors compared to controls (Fig. 1k). Immunohistochemistry staining for γH2AX revealed a significantly higher percentage of γH2AX-positive tumor cells in PARPi-treated *FANCA*-deficient tumors, implicating DNA damage accumulation as a likely driver of tumor cell synthetic lethality (Fig. 1l).

Notably, the observed synthetic lethality between PARPi and *FANCA* deficiency was not restricted to breast cancer models. CRISPR-mediated *FANCA*-knockout in the lung cancer cell line models A549 and H1299 also sensitized cells to PARPi, demonstrating that *FANCA* deficiency induces synthetic lethality across multiple cancer types (Extended Data Fig. 1i-l). Collectively, these findings establish *FANCA* deficiency as a potent determinant of PARPi sensitivity across *in vitro* and *in vivo* human isogenic cancer models.

### A FANCA-FAAP20 subcomplex mediates PARPi response

*FANCA* is the most frequently mutated gene in Fanconi Anemia, encoding a protein that forms part of the FA core complex responsible for mediating FANCD2 ubiquitylation during ICL repair. Since FANCA is a non-essential component of the FA core complex for *in vitro* FANCD2 ubiquitylation^21^, we sought to clarify whether deficiencies in other components of the FA pathway would similarly induce PARPi SL. Towards this end, we generated isogenic MDA-MB-231 clones deficient in *FANCM* and *FANCD2* (Extended Data Fig. 2a). However, neither *FANCM*- nor *FANCD2*-deficient clones exhibited a level of PARPi SL comparable to that seen with *FANCA* deficiency (Extended Data Fig. 2b). Given that FANCA forms a subcomplex with FANCG and FAAP20^17,20^, we further evaluated the phenotypes of cells lacking these components. Interestingly, FAAP20 deficiency, but not FANCG deficiency, recapitulated the PARPi SL observed in *FANCA*-deficient cells (Extended Data Fig. 2c,d). Because FANCG mediates the interaction between FANCA and the FA core complex^41^, our findings suggest that PARPi SL arises not from defects in canonical FA pathway-mediated FANCD2 ubiquitylation, but rather from the disruption of a distinct molecular function mediated by the FANCA-FAAP20 subcomplex.

### FANCA deficiency does not impair HR repair

**Figure 2:**
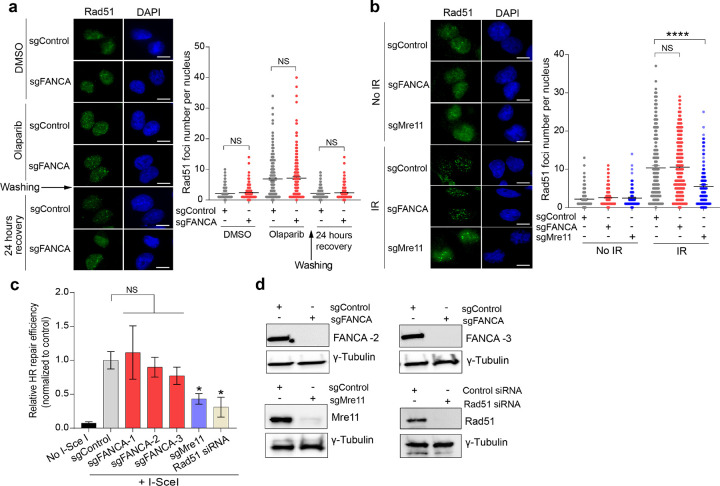
*FANCA* deficiency does not impair homologous recombination (HR) repair. **a,** Representative immunofluorescence (IF) images of Rad51 foci in MDA-MB 231 cells with the indicated genotypes after Olaparib treatment (10 μM, 4 hours), followed by a 24-hour recovery. Quantification of Rad51 foci per nucleus was performed using ImageJ. Data are presented as mean ± SEM (n=3 biologically independent experiments), analyzed by an unpaired, two-tailed t-test. **b,** IF analysis of Rad51 foci in MDA-MB-231 cells with the indicated genotypes 4 hours after IR (8Gy). Quantified data (mean ± SEM, n=3 biologically independent experiments) were analyzed using an unpaired, two-tailed t-test; ****p<0.0001. **c,** HR repair efficiency was assessed in pDR-GFP MDA-MB-231 cells with the indicated genotypes. Cells were transfected with either I-SceI or an empty vector for 48 hours, followed by flow cytometry analysis of GFP-positive cells. Data are shown as mean± SEM (n=3 biologically independent experiments), analyzed by an unpaired, two-tailed t-test; *p<0.05. Scale bars, 10 μm.

The FA pathway mediates ICL repair through downstream recruitment of TLS and HR pathways. To determine whether *FANCA* deficiency attenuates HR repair, we first assessed RAD51 foci formation, a hallmark of HR repair activity. Using independent *FANCA*-knockout clones for both MDA-MB-231 and BT-549 cell lines, we observed no significant differences in RAD51 foci formation following 4-hour Olaparib treatment and 24-hour recovery, compared to control cells (Fig. 2a and Extended Data Fig. 3a,b). Radiation treatment similarly revealed no differences in RAD51 foci formation between *FANCA*-deficient and control cells, whereas *MRE11*-mutant cells (a positive control) displayed markedly reduced RAD51 foci, consistent with the established role of Mre11 in promoting DNA end resection, which is essential for Rad51 loading (Fig. 2b)^42,43^.

To further examine HR repair efficiency, we performed the DR-GFP reporter assay, a widely used method for measuring HR-dependent DSB repair^44^. Three independent *FANCA*-knockout clones exhibited HR activity comparable to that of controls, whereas cells expressing a *MRE11* hypomorphic mutation or *RAD51* knockdown (positive controls) showed significantly impaired HR activity (Fig. 2c,d). Together, these results establish that *FANCA* deficiency does not disrupt HR-mediated DSB repair, indicating that PARPi SL is induced through a HR-independent mechanism.

### FANCA deficiency induces ssDNA accumulation and RPA exhaustion after PARPi treatment

**Figure 3:**
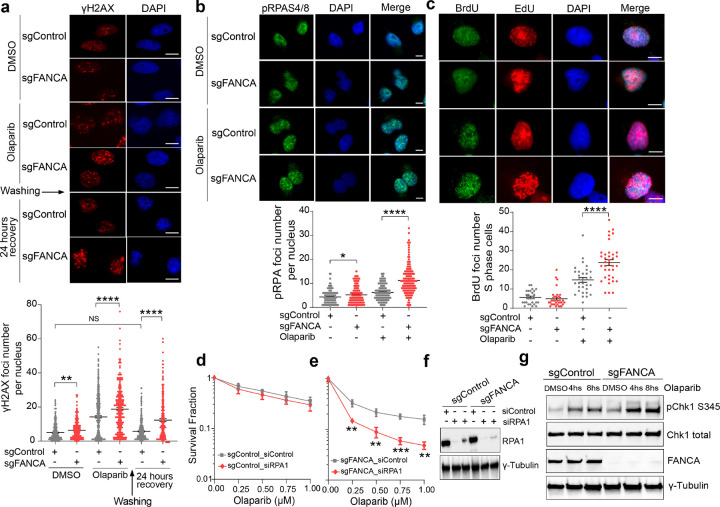
FANCA suppresses ssDNA accumulation after PARPi treatment. **a,** Representative IF images of gH2AX foci in MDA-MB-231 cells with the indicated genotypes, either untreated or treated with Olaparib (10 μM, 4 hours), followed by a 24-hour recovery. Quantification of gH2AX foci per nucleus was performed. Data represent mean ± SEM (n=3 biologically independent experiments). Significance was assessed using an unpaired, two-tailed t-test; **p<0.01, ****p<0.0001. **b, c,** IF analysis of pRPA S4/8 foci (**b**) and BrdU-labeled ssDNA foci (**c**) in MDA-MB-231 cells with the indicated genotypes, either untreated or treated with Olaparib (10 μM, 4 hours). Representative images and quantified data are shown. Data are presented as mean± SEM from three (**b**) and two (**c**) biologically independent experiments; *p<0.05, ****p<0.0001 (unpaired two-tailed t-test). **d, e,** RPA1 depletion reduces cell survival. MDA-MB-231 cells with the indicated genotypes were transfected with either control siRNA or RPA1-targeting siRNA for 48 hours, followed by colony formation assays in the presence of Olaparib. Data represent mean ± SEM (n=4 biologically independent replicates); **p<0.01, ***p<0.001 (unpaired, two-tailed t-test). **f,** Western blot analysis confirmed RPA1 depletion in cells transfected with RPA1 siRNA. **g,** Increased Chk1 phosphorylation (pChk1 S345) was observed in *FANCA*-deficient MDA-MB-231 cells. Western blot analysis was performed to examine pChk1, total Chk1, and FANCA levels in the indicated genotypes, either untreated or treated with Olaparib (10 μM) at indicated time points. Scale bars, 10 μm.

Having demonstrated that FANCA is not required for HR-mediated DSB repair, we hypothesized that *FANCA* deficiency might affect the cellular response to ssDNA damage. To investigate this, we broadly assessed DNA damage by performing γH2AX immunofluorescence. Kinetic analysis of γH2AX foci in the TNBC cell lines MDA-MB-231 and BT549 revealed significant increases in unrepaired DNA damage in *FANCA*-deficient cells 4 hours after PARPi treatment, with persistently elevated foci levels after 24-hour recovery (Fig. 3a and Extended Data Fig. 3c). Interestingly, even in the absence of PARPi, *FANCA*-deficient cells exhibited modestly elevated γH2AX foci, suggesting that *FANCA* deficiency alone may be sufficient to increase DNA damage accumulation.

Given recent studies implicating PARP1 in resolving daughter strand ssDNA gaps, we next examined ssDNA accumulation^27,28,33,45^. *FANCA*-deficient clones exhibited significantly increased levels of pRPAS4/8 foci and native BrdU foci in S phase cells following PARPi treatment (Fig. 3b,c). RPA is essential for protecting ssDNA, and prior studies have indicated that ssDNA accumulation can induce cellular lethality due to an exhaustion of cellular RPA levels^33,46^. Based on these observations, we reduced the available RPA pool using siRNA or a small-molecule RPA inhibitor (RPAi) and found that this exacerbated the synthetic lethality associated with *FANCA* deficiency and PARPi (Fig. 3d–f and Extended Data Fig. 4a,b). These findings identify ssDNA accumulation and RPA exhaustion as key drivers of the observed lethality.

Consistent with a deficiency in the replication stress response, *FANCA*-deficient cells also demonstrated significant sensitivity to hydroxyurea (HU) and aphidicolin (APH) (Extended Data Fig. 5a,b). Treatment with HU and APH also increased γH2AX and pRPAS4/8 foci levels in *FANCA*-deficient cells, implicating FANCA in a pathway that mitigates ssDNA accumulation in response to diverse forms of replication stress (Extended Data Fig. 5c,d). Additionally, *FANCA*-deficient cells showed elevated pCHK1 levels compared to control cells after PARPi treatment (Fig. 3g), indicating that ssDNA accumulation in this context triggers heightened ATR signaling.

### FANCA deficiency impairs Okazaki fragment maturation (OFM)

**Figure 4:**
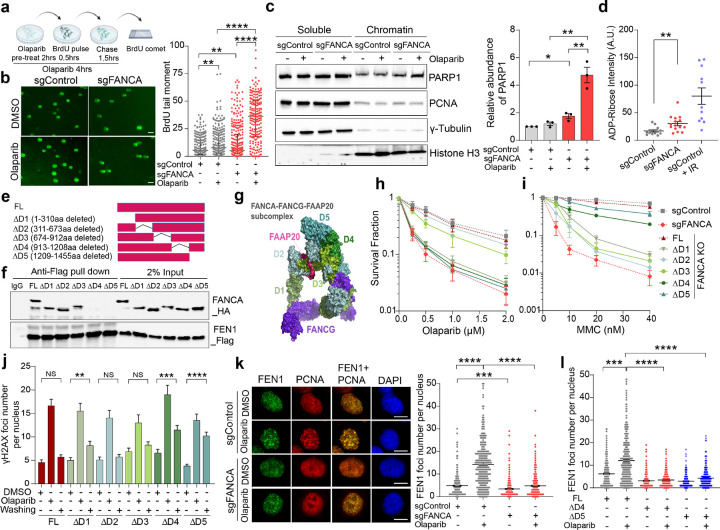
The FANCA C-terminal domain is required for FEN1 recruitment and Okazaki fragment maturation. **a, b,** *FANCA* deficiency impairs nascent DNA strand integrity. **a,** Schematic of the BrdU comet assay protocol. MDA-MB-231 cells with the indicated genotypes were either untreated or treated with Olaparib (10 μM). After BrdU pulse-labeling and chase, nascent single strands were assessed using anti-BrdU antibody staining in alkaline comet assays. **b,** Representative images (left) and quantification of BrdU+ comet tail moments (right) are shown. Data are presented as median ± interquartile range (n=3 biologically independent experiments), analyzed by unpaired, two-tailed t-test. **p<0.01, ****p<0.0001. Scale bars, 50 μm. **c,** *FANCA* deficiency increases PARP1 chromatin association. MDA-MB-231 cells with the indicated genotypes were untreated or treated with Olaparib (10 μM) for 4 hours, followed by nuclear fractionation. Western blot analysis (left) quantified PARP1 levels in chromatin, normalized to histone H3 (right). Data are mean ± SEM (n=3 independent biological replicates), analyzed by unpaired, two-tailed t-test. *p<0.05, **p<0.001. d, FANCA deficiency increases ADP-ribosylation. ADP-ribose was detected by IF staining using ADP-ribose (MAR/PAR) monoclonal antibody in MDA-MB-231 cells with the indicated genotypes with 2.5 mM hydroxyurea (HU) treatment for 20 hours. The IR-treated sample served as a positive control. Data shown are mean ± SEM, and statistical significance was assessed by unpaired two-tailed t-test. **p<0.01. **e,f,** The C-terminal domains (D4 and D5) of FANCA mediate FEN1 interaction. **e,** Schematic illustration of full-length (FL) and deletion mutants of FANCA used in this study. FANCA constructs were cloned into inducible PiggyBac expression vectors. **f,** Co-IP experiment with anti-Flag antibody to pull down Flag-tagged proteins using whole cell lysate and Western blot were performed as indicated. **g,** FANCA-FANCG-FAAP20 subcomplex cryoEM structure model (PDB #7KZP) with color-coded D1 – D5 subdomains. **h,i** Functional relevance of FANCA domains. Clonogenic survival assays were conducted on *FANCA*^*−/−*^ MDA-MB-231 cells reconstituted with FANCA FL or DD1-D5 mutants treated with Olaparib (**h**) or mitomycin C (**i**). Data are mean ± SEM (n=3 biologically independent replicates). **j,** FANCA DD4 and DD5 mutants impair PARPi-induced DNA damage repair. IF analysis of gH2AX foci in *FANCA*^*−/−*^ MDA-MB-231 cells reconstituted with FL or DD1-D5 mutants, either untreated or treated with Olaparib (10 μM) for 4 hours, followed by 24-hour recovery. Data are mean ± SEM, analyzed by unpaired, two-tailed t-test. **p<0.01, ***p<0.001, **** p<0.0001. **k,l** FANCA promotes FEN1 recruitment. (**k**) Representative IF images of FEN1 and PCNA in MDA-MB-231 cells with the indicated genotypes, untreated or treated with Olaparib (10 μM) for 4 hours. Quantification of FEN1 foci (right) is shown. Data are mean ± SEM (n=4 biologically independent experiments), analyzed by unpaired two-tailed t-test. ***p<0.001, **** p<0.0001. Scale bars, 10 μm. **l,** IF analysis of FEN1 foci in *FANCA*^*−/−*^ MDA-MB-231 cells reconstituted with FL, ΔD4, or ΔD5 mutants under the same conditions. Data are mean ± SEM (n=3 biologically independent experiments), analyzed by unpaired two-tailed t-test, ***p<0.001, **** p<0.0001. ΔD1- ΔD5: deleted domains 1–5.

OFM is critical for completion of lagging strand synthesis during DNA replication^30, 47^. Defective OFM can generate cytotoxic ssDNA daughter strand gaps, particularly under replication stress^29,30,47^. Prior studies have demonstrated an essential role for PARP1 in recognizing nascent lagging strand gaps to stimulate a XRCC1- and LIG3-dependent alternative pathway for OFM^27,28,48^. Based on our observations, we postulated that an OFM impairment may underlie synthetic lethality of *FANCA*-deficient cells to PARPi. To test this hypothesis, we first assessed single- and double-stranded DNA breaks using an alkaline comet assay, which revealed significantly more endogenous DNA breaks in *FANCA*-deficient cells, further exacerbated by PARPi treatment (Extended Data Fig. 6a). To specifically evaluate nascent strand integrity during OFM, we performed BrdU comet assays^29^, where BrdU positive comet tails quantify unligated nascent DNA strands (Fig. 4a). *FANCA*-deficient cells exhibited significantly more unligated nascent DNA strands compared to controls, indicating FANCA is required for OFM, with PARPi treatment significantly exacerbating this phenotype (Fig. 4b).

Additionally, *FANCA*-deficient cells demonstrated increased PARP1 levels in the detergent-insoluble chromatin fraction, particularly after PARPi treatment (Fig. 4c). These findings suggest the accumulation of incompletely processed Okazaki fragments in *FANCA*-deficient cells, as PARP1 is known to act as a sensor and binder of unligated Okazaki fragments^27,28^. Consistent with elevated PARP1 engagement during DNA replication stress, *FANCA*-deficient cells displayed increased poly(ADP-ribose) (PAR) levels following HU treatment, with or without poly(ADP-ribose) glycohydrolase (PARG) inhibition, which blocks a major PAR degradation pathway^28,29,49^ (Fig. 4d and Extended Data Fig. 6b). Collectively, these data suggest a role for FANCA in OFM, where its impairment necessitates PARP1-dependent daughter strand gap repair mechanisms for alternative OFM. Consequently, PARPi treatment in *FANCA*-deficient cells leads to substantial accumulation of unligated nascent Okazaki fragments.

### FANCA promotes OFM through physical interaction with FEN1

Prior biochemical studies have shown that FANCA preferentially binds 5’ DNA and RNA flap structures and stimulates FEN1 flap endonuclease activity, providing a potential mechanistic linkage to OFM^24,50,51^. Consistent with this, we observed a physical interaction between FANCA and FEN1 in reciprocal co-immunoprecipitation (co-IP) experiments using 293T cells co-expressing FANCA and FEN1 (Extended Data Fig. 7a). To further define the domains critical for this interaction, we generated a series of HA-tagged FANCA deletion mutants (*FANCA* ΔD1 to ΔD5)^20^, each lacking approximately 300 amino acids, and used a doxycycline-inducible system overexpress these mutants (Fig. 4e and Extended Data Fig. 7b).

Co-IP analysis with Flag-tagged FEN1 revealed that *FANCA* ΔD4 and ΔD5 mutants failed to interact with FEN1 (Fig. 4f), identifying the FANCA C-terminal region (amino acids 913–1455) as essential for FEN1 binding. Notably, this region is on the opposite surface from FANCG interaction in the FANCA-FANCG-FAAP20 subcomplex^52^, further corroborating our earlier observation that FANCG and the FA core complex are not required for this FANCA-specific function (Fig. 4g).

We next hypothesized that the FANCA-FEN1 interaction is crucial for FANCA’s role in PARPi resistance. In *FANCA*-deficient cells, reconstitution with full-length *FANCA* rescued clonogenic survival following PARPi treatment (Extended Data Fig. 7c and Fig. 4h). However, *FANCA* ΔD1, ΔD4, or ΔD5 mutants failed to restore survival, while *FANCA* ΔD2 and ΔD3 mutants fully rescued PARPi resistance (Fig. 4h). In contrast, reconstitution of *FANCA*-deficient cells with *FANCA* ΔD4 or ΔD5 mutants restored cell survival following the ICL-inducing agent MMC, whereas *FANCA* ΔD2 and ΔD3 mutants did not (Fig. 4i). This indicates a bifunctional role for FANCA: its C-terminal region mediates PARPi resistance, while its middle region is required for canonical ICL repair through interaction with FANCG and the FA core complex. The *FANCA* ΔD1 mutant likely disrupts the nuclear localization signal of FANCA^17,53^, leading to deficiencies in both molecular functions.

We further examined γH2AX foci as a marker of unresolved DNA damage in *FANCA*-reconstituted cells. In cells reconstituted with full-length *FANCA,* γH2AX foci resolved to basal levels 24 hours after PARPi treatment. In contrast, *FANCA* ΔD4 and ΔD5 mutant-reconstituted cells exhibited persistent γH2AX foci (Fig. 4j). To evaluate whether FANCA may contribute to FEN1 recruitment during OFM, we examined chromatin recruitment of FEN1 following PARPi treatment. FEN1 chromatin recruitment was significantly increased after PARPi treatment in *FANCA*-proficient cells but abolished in *FANCA* deficient cells across both MDA-MB-231 and BT549 cells (Fig. 4k and Extended Data Fig. 7d). Reconstitution with full-length *FANCA* restored PARPi-induced FEN1 recruitment, whereas *FANCA* ΔD4 and ΔD5 mutants remained defective (Fig. 4l). These results demonstrate that the C-terminal region of FANCA is required for enhanced FEN1 recruitment to chromatin upon PARP1 inhibition.

### Oncogene expression drives synthetic lethality of FANCA deficiency with PARPi

**Figure 5:**
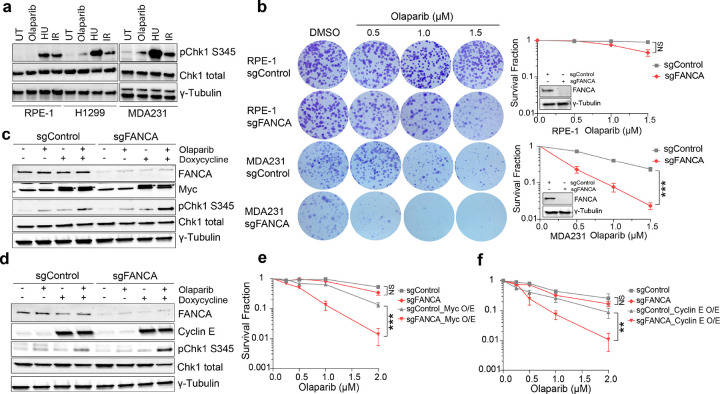
Oncogenic stress is required for FANCA deficiency-driven synthetic lethality with PARPi. **a,** Differential ATR signaling response to Olaparib. hTERT RPE-1, MDA-MB-231, and H1299 cells were treated with Olaparib (10μM) for 4 hours. Western blot analysis measured Chk1 phosphorylation at Ser345. HU- and IR-treated samples served as positive controls. **b,** Clonogenic survival assays for hTERT RPE-1 and MDA-MB-231 cells with the indicated genotypes after Olaparib treatment. Insets show Western blot confirmation of *FANCA* deficiency in the corresponding cell lines. Data are mean ± SEM (n=3 biologically independent replicates), analyzed by unpaired two-tailed t-test. ***p<0.001. **c,d,** Oncogene expression activates ssDNA gap-induced ATR signaling. Inducible PiggyBac vectors expressing (**c**) c-Myc or (**d**) Cyclin E were constructed as described in Methods. Cells were treated with 200 ng/ml doxycycline for 24 hours to induce oncogene expression, followed by Olaparib treatment (10μM) for 4 hours. Western blot analysis was performed with the indicated antibodies. **e, f,** Oncogenic c-Myc or Cyclin E is essential for PARPi-induced cell death in *FANCA*-deficient cells following Olaparib treatment. CFAs were conducted in hTERT RPE-1 cells with the indicated genotypes expressing c-Myc (**e**) or Cyclin E (**f**). Data are mean ± SEM (n=3 biological independent replicates), analyzed by unpaired two-tailed t-test at the highest drug dosage., **p<0.01, ***p<0.001. * Myc band.

Oncogene overexpression in cancer cells induces replication stress and chronically elevated DNA damage levels^54–56^. To investigate whether oncogene-driven replication stress promotes the synthetic lethality of *FANCA* deficiency with PARPi, we examined ATR signaling activation in non-cancerous (hTERT RPE-1) and malignant (MDA-MB-231, H1299) cells following PARPi treatment. ATR signaling, measured by pChk1-S345 levels, was significantly elevated in cancer cells after PARPi treatment but nearly undetectable in non-transformed hTERT RPE-1 cells (Fig. 5a). This suggests that oncogene-induced replication stress may promote PARPi-associated SL. Clonogenic survival assays confirmed this hypothesis: *FANCA* deficiency significantly sensitized MDA-MB-231 cells to PARPi, while in hTERT RPE-1 cells, *FANCA* deficiency caused only a modest increase in PARPi-associated cell lethality (Fig. 5b).

To further dissect this effect, we generated a doxycycline-inducible system for c-Myc or Cyclin E overexpression in hTERT RPE-1 and MDA-MB-231 cells (Extended Data Fig. 8a,b). In both cell types, induction of c-Myc or Cyclin E resulted in higher ATR activation (pChk1-S345) following PARPi treatment, which was absent in controls (Extended Data Fig. 8c,d). Notably, *FANCA*-deficient hTERT RPE-1 cells overexpressing c-Myc or Cyclin E exhibited the greatest increase in pChk1-S345 levels, suggesting that oncogene overexpression may amplify the synthetic lethality between *FANCA* deficiency and PARPi (Fig. 5c,d). Clonogenic survival assays further demonstrated that c-Myc or Cyclin E overexpression was sufficient to restore synthetic lethality between *FANCA* deficiency and PARPi in hTERT-RPE cells, when compared to non-oncogene expressing controls (Fig. 5e,f). Collectively, these observations reveal that FANCA-PARPi synthetic lethality manifests only in the context of oncogene-induced replication stress.

**Figure 6:**
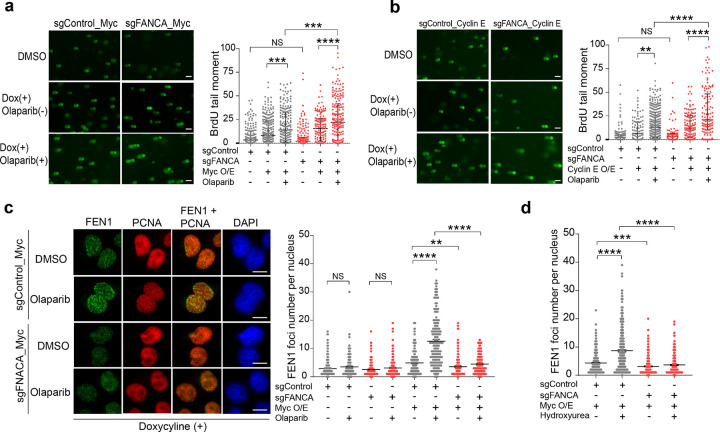
Oncogene expression combined with FANCA deficiency compromises OFM and FEN1 recruitment following PARPi treatment. **a,b,** BrdU comet assays were performed on hTERT RPE-1 sgControl or sgFANCA cells with doxycycline-inducible (**a**) c-Myc (sgControl_Myc or sgFANCA_Myc) or (**b**) Cyclin E (sgControl_Cyclin E or sgFANCA_Cyclin E) expression, either untreated or treated with Olaparib (10 μM) for 4 hours. Representative comet images (left) and quantification of comet tail moment (right) are shown. Data represent the median ± interquartile range across two biologically independent experiments, analyzed by unpaired two-tailed t-test. ** p<0.01, ***p<0.001, **** p<0.0001. Scale bars, 50 μm. **c,** Representative IF images of FEN1 and PCNA in hTERT RPE-1 cells with the indicated genotypes under doxycycline induction and either untreated or treated with Olaparib for 4 hours (left). FEN1 foci were quantified and plotted (right). Data shown are mean ± SEM, pooled from three biologically independent experiments, and analyzed by unpaired two-tailed t-test. **p<0.01, **** p<0.0001. Scale bars, 10 μm. **d,** IF analysis of FEN1 foci in the indicated hTERT RPE-1 genotype cells under doxycycline induction (200 ng/ml) either untreated or treated with HU (250 nM) for 3 hours. Data are mean ± SEM (n= 2 biologically independent experiments), and analyzed by unpaired two-tailed t-test. **p<0.01, **** p<0.0001.

BrdU comet assays further confirmed the role of oncogene-induced replication stress in priming cells for Okazaki fragment maturation defects. *FANCA*-deficient cells overexpressing c-Myc or Cyclin E exhibited significantly greater impairment of nascent Okazaki fragment maturation after PARPi treatment compared to control cells (Fig. 6a,b). Furthermore, oncogene expression was required for FANCA-dependent induction of FEN1 recruitment during PARPi treatment, which was significantly reduced by *FANCA* deficiency (Fig. 6c). This effect could be recapitulated by HU treatment (Fig. 6d), indicating that oncogenic stress induces FANCA-dependent FEN1 recruitment as a mechanistic basis for synthetic lethality.

### R-loops mediate oncogene-driven FANCA-PARPi synthetic lethality

**Figure 7:**
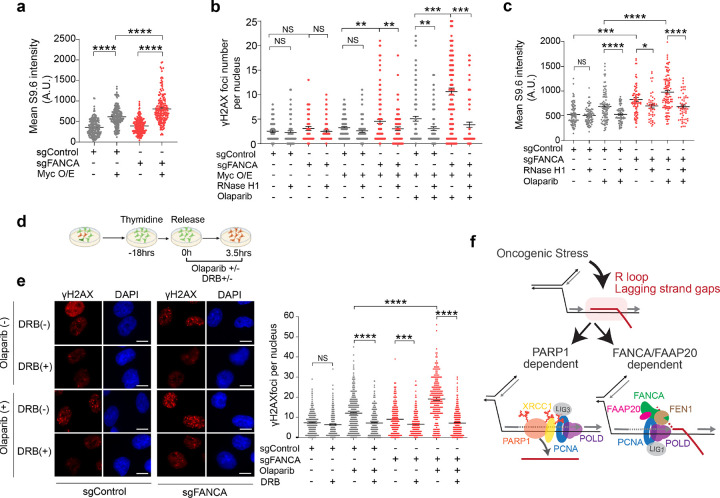
Oncogenic stress-induced R-loop accumulation drives the FANCA-PARPi synthetic lethality. **a,** IF staining of R-loops using the S9.6 antibody was performed in the indicated hTERT RPE-1 cells. R-loop intensity per cell was quantified by ImageJ. Data represent mean ± SEM (n=3 biologically independent experiments) and were analyzed by unpaired two-tailed t-test. **** p<0.0001. **b,** IF analysis of gH2AX foci in the indicated hTERT RPE-1 cells. Cells were transfected with an RNase H1 construct for one day, followed by treatment with Olaparib (10 μM) for 4 hours or no treatment. Doxycycline (200 ng/ml) was used to induce c-Myc expression. Data represent mean ± SEM, analyzed by an unpaired, two-tailed t-test. **p < 0.01, ***p < 0.001. **c,** IF staining of R-loops was conducted in the indicated MDA-MB-231 cells. Cells were transfected with an RNaseH1 construct for one day, followed by treatment with Olaparib (10 μM) for 4 hours or no treatment. R-loop intensity was quantified by ImageJ. Data represent mean ± SEM (n=2 biologically independent experiments), assessed by an unpaired, two-tailed t-test. * p<0.05, ***p<0.001, **** p<0.0001. **d,** Schematic representation of the experimental procedure for DRB treatment. **e,** The indicated MDA-MB-231 cells were synchronized with 2 mM thymidine for 18 hours and released into fresh medium containing either Olaparib (10 μM) and/or DRB (75 μM) for 3.5 hours, as indicated. Representative IF images of gH2AX foci are shown (left). Quantification of the gH2AX foci number is plotted (right). Data represent mean ± SEM (n=3 biologically independent experiments) and were analyzed by unpaired, two-tailed t-test. ***p<0.001, **** p<0.0001. Scale bars, 10 μm. **f,** Proposed model illustrating the role of FANCA in FEN1-mediated RNA flap cleavage during Okazaki fragment maturation in the presence of oncogene-induced post-replication R-loops on the lagging strand. DRB: 5,6-Dichloro-1-β-D-ribofuranosylbenzimidazole.

The vast transcriptional programs induced by oncogenes increase the frequency of transcription-replication conflicts (TRCs), which have recently been implicated as drivers of PARPi-mediated synthetic lethality^57^. We hypothesized that oncogene-induced TRCs, manifested as transcription-associated RNA-DNA hybrid (R-loop) structures, might similarly contribute to FANCA-PARPi SL by impeding OFM. To test this, we stained for R-loops using the S9.6 antibody and observed significant R-loop accumulation in hTERT RPE-1 cells overexpressing c-Myc, which was further exacerbated by *FANCA* deficiency (Fig. 7a and Extended Data Fig. 9a).

To evaluate the causal role of R-loops in DNA damage accumulation, we overexpressed RNase H1 to degrade RNA-DNA hybrids, and assessed DNA damage after PARPi treatment. In *FANCA*-deficient cells overexpressing c-Myc, RNase H1 expression markedly reduced γ-H2AX foci both with or without PARPi treatment (Fig. 7b and Extended Data Fig. 9b,c). Similar findings were observed in MDA-MB-231 cells, where *FANCA* depletion increased R-loop abundance, which was further amplified following PARPi treatment and fully reversed by RNase H1 co-expression (Fig. 7c and Extended Data Fig. 9d).

To distinguish stable R-loop structures from those arising during transcription, we synchronized cells in S phase and treated them with the RNA polymerase II elongation inhibitor, 5,6-Dichloro-1-β-D-ribofuranosylbenzimidazole (DRB). DRB suppressed DNA damage accumulation in *FANCA*-deficient MDA-MB-231 and BT549 cells after treatment with PARPi (Fig. 7d,e and Extended Data Fig. 9e). These findings suggest that oncogene-induced co-transcriptional R-loops are stabilized by *FANCA* deficiency and PARPi treatment, and thus drive the accumulation of unligated Okazaki fragments and catastrophic DNA damage that underlies synthetic lethality.

## Discussion

Our study illustrates the promise of *in vivo* CRISPR tumorigenesis screens coupled with pooled therapeutic selection to uncover novel SL interactions enriched for tumor-promoting variants. This approach addresses a key limitation of *in vitro* CRISPR screens that has hampered the clinical translation of SL-directed therapies. Specifically, we identify *FANCA* deficiency as a clinically relevant SL interaction with PARPi due to impaired lagging strand DNA synthesis—a mechanism distinct from canonical HR deficiency as a basis for PARPi SL. These findings expand the potential clinical utility of PARPi.

In cells expressing oncogenes, *FANCA* deficiency combined with PARPi treatment induces a lethal accumulation of DNA replication-associated ssDNA gaps and RPA exhaustion. This oncogene-specific dependency explains why prior analyses of PARPi sensitivity in immortalized FA mutant cells failed to reveal significant SL interactions between *FANCA* deficiency and PARPi^58^. In our isogenic models, deficiencies in *FANCG, FANCM,* or *FANCD2* did not replicate the PARPi SL phenotype seen with *FANCA* deficiency, implicating a critical novel role for FANCA in promoting PARPi resistance. However, deficiency in FAAP20, a protein that contains a ubiquitin-binding zinc-finger (UBZ) domain that is required for DNA damage-induced chromatin loading of FANCA^59^, phenocopied the PARPi SL observed with FANCA loss. Thus, our findings identify the FANCA-FAAP20 subcomplex as essential for PARPi resistance under oncogenic stress.

OFM is essential for DNA replication, and its disruption can result in a potentially lethal accumulation of lagging strand gaps. We demonstrate that *FANCA* deficiency combined with PARPi significantly impairs OFM, leading to ssDNA accumulation and RPA exhaustion. Recent evidence has elucidated multiple OFM pathways with distinct molecular mediators. Canonical OFM is mediated by PCNA, FEN1, and LIG1, whereas an alternative pathway relies on PARP1, XRCC1, and LIG3^27,28^. Our findings establish FANCA as a promoter of canonical OFM, enhancing FEN1 recruitment during oncogene-driven replication stress or nucleotide depletion (e.g., after HU treatment). Additionally, FANCA is essential for the increased chromatin recruitment of FEN1 in response to PARPi, underscoring the delicate balance between FEN1- and PARP1-mediated OFM pathways during replication stress.

Our genetic and biochemical analyses reveal that the C-terminal region of FANCA uniquely facilitates FEN1 recruitment during OFM, providing an *in vivo* context and significance for FANCA’s previously reported enhancement of FEN1 nucleolytic activity^24^. This role is genetically separable from its canonical function in ICL repair via the FA pathway. FANCA’s preferential binding to single-stranded flap structures^50^ may optimally position it to facilitate more efficient FEN1 recruitment and nucleolytic processing, particularly during heightened replication stress when cellular FEN1 levels may become rate-limiting. Together, our findings underscore FANCA's critical role in FEN1 recruitment and activity during OFM under oncogene-associated replication stress.

*FANCA* deficiency promotes engagement of an alternative OFM pathway that is PARP1-dependent. Recent studies have demonstrated that an alternative pathway entails PARP1-mediated LIG3 recruitment to anneal Okazaki fragments independently of FEN1 and LIG1^38,60,61^. The fork protection complex mediator TIMELESS has been shown to interact with PARP1 and is required for engagement of PARP1-dependent alternative OFM^45,57,62^. Thus, the increased utilization of PARP1-dependent OFM upon *FANCA* deficiency may confer a growth advantage to cancers with high levels of oncogene-induced replication stress where rate-limiting levels of FEN1 and LIG1 may limit cell cycle progression, potentially explaining the relatively high rate of *FANCA* genetic alterations in pan-cancer cohorts (Fig. 1g). Our study reveals a therapeutic vulnerability of such cancers to PARPi treatment, which induces an accumulation of unligated Okazaki fragments, RPA exhaustion, and cellular lethality.

Our findings indicate that oncogene-induced co-transcriptional R-loops are a major driver of the synthetic lethality observed between *FANCA* deficiency and PARPi. This aligns with similar observations in HR-deficient contexts, where TRCs and R-loops play a prominent role in driving PARP1 dependency^63,64^. While the precise nature of these R-loops remains to be better defined, recent evidence suggests that R-loops may preferentially form on the lagging strand during DNA replication, leading to daughter strand gap accumulation and creating a physical barrier to OFM^65–67^. Notably, both PARP1 and FANCA have been implicated in resolving co-transcriptional R-loops, which our data suggests may be the primary substrate underlying their synthetic lethality in OFM (Fig. 7f)^63,68^. Lagging strand DNA synthesis may generate single-stranded 5’ RNA flaps, which are a preferential substrate for FANCA binding^50^. FANCA-mediated recruitment of FEN1 to these sites—particularly during PARPi treatment—may facilitate R-loop resolution, consistent with the established ability of FEN1 to cleave RNA flaps^69^. Our results, in combination with other recent studies, suggest that oncogene-induced replication stress increases the burden of post-replication lagging strand R-loops, necessitating FANCA-dependent FEN1 recruitment, PARP1-dependent alternative OFM, and/or HR repair to support cellular viability. Deficiency in either *FANCA* or HR creates a critical dependency on PARP1-dependent alternative OFM, thereby inducing PARPi synthetic lethality. Further work is needed to better characterize the oncogene-induced TRCs underlying these phenotypes, such as their directionality (co-directional vs. head-on) and genome-wide distribution. Additionally, the accumulation of R-loops in underreplicated regions may stimulate immune signaling^70,71^, presenting additional opportunities to enhance the therapeutic efficacy of PARPi through combination strategies.

Our study establishes *FANCA* genetic alterations in cancer as a promising selection criterion for PARP1 inhibitor treatment. Given that *FANCA* mutation prevalence is comparable to *BRCA1* and *BRCA2* mutations, this may significantly increase the clinical applicability of PARP1 inhibitors, particularly when also considering other hits identified in our *in vivo* screen (Fig. 1b). The conditional dependency on oncogene-driven replication stress for FANCA-PARPi synthetic lethality suggests that combining PARPi with agents that elevate replication stress, such as ATR or CHK1 inhibitors, could further enhance therapeutic efficacy. Notably, our findings also suggest that PARPi may also demonstrate a therapeutic window in Fanconi Anemia patients with biallelic *FANCA* mutations who develop cancer—often associated with Myc amplification and replication stress-associated genomic instability^72,73^—providing a targeted therapy for a rare but challenging clinical scenario.

## Methods

### Cell line Culture and Gene Editing

Human MDA-MB-231 cells were grown in MEM medium supplemented with 10% FBS and 1% sodium pyruvate. hTERT-RPE1 cells were grown in DMEM + GlutaMAX-I medium supplemented with 10% FBS. A549 cells were cultured in F-12K medium supplemented with 10% FBS. H1299 and BT549 cells were maintained in RPMI 1640 medium supplemented with 10% FBS. All cell lines were cultured at 37 °C with 5% CO_2_. FANCA, FANCD2, FANCM, FANCG, FAAP20, or BRCA1 gene knockout in MDA-MB-231 cells was induced using three synthesized sgRNAs for each line, purchased from Synthego (FANCA sgRNA#1 CCGGCGAAACCGUCCCGGGC, sgRNA#2 CCUACCCAGCAGCUCGGCCC, sgRNA#3 UGGGUCCCGAACUCCGCCUC; FANCD2 sgRNA#1 GAUCAGCUGCAUGAUCUUGG, sgRNA#2 ACCAGCCUACCUGAGAUCCU, sgRNA#3 CCUCACUUAGAUGAUGACAG; FANCM sgRNA#1 GCUCUGAGGUCGCUCAGUUC, sgRNA#2 CGAUGAUGUGUUGCUUGUCG, sgRNA#3 UGGCGGGUUCUGCACCUCCG; FANCG sgRNA#1 GAAGAACAGGAACAGCUGCA, sgRNA#2 CACCUCUCUCUAGGCUCCGC, sgRNA#3 CUGAGGGCAAGCUUGGCCCA; FAAP20 sgRNA#1 GUGUUGCAGGAGCCCAGGUG, sgRNA#2 UUCCUGGACACCCUUUCCGC, sgRNA#3 CUCCUGCCCCGUGAAGCAGC; BRCA1 sgRNA#1 CUGCUAUUUAGUGUUAUCCA, sgRNA#2 UCCAUUCUUUUCUCUCACAC, sgRNA#3 GUAAGGAAACAUGUAAUGAU). Non-targeting sgRNA control was also used (GCACUACCAGAGCUAACUC). FANCA gene knockout in A549, H1299, and BT549 cells was also induced using the same FANCA sgRNAs listed above. Briefly, 40 pmol of sgRNA and 10 pmol of Cas9 were mixed for 10 minutes at room temperature to form the RNP complex which was then introduced into cells via the Neon electroporation system. Two days later, 40 electroporated cells were added in 25 ml of the corresponding complete medium and dispersed into one 96-well plate. At least 15 single-cell clones were picked up after two weeks of incubation and transferred to a six-well plate for further cell growth. A western blot was subsequently performed to confirm the knockout efficiency of each target gene.

### Chemicals, antibodies, siRNAs, and plasmids

PARP inhibitor Olaparib (AZD-2281, SelleckChem, cat. no. S1060), ATR inhibitor (Ceralasertib, SelleckChem, cat. no. S7693), PARG inhibitor (SelleckChem, cat. no. S8862), Mitomycin C (MilliporeSigma, cat. no. M4287), RPA inhibitor (TDRL-551, MedChemExpress, cat. no. HY-114842), BrdU (MilliporeSigma, cat. no. B9285), 5,6-Dichloro-1-β-D-ribofuranosylbenzimidazole (DRB) (Millipore Sigma, cat. no. D1916). Concentration and treatment duration of the chemicals are described in the figures and sections correspondingly.

The antibodies and dilutions used in this study were as follows: anti-FANCA (Cell Signaling, cat. no. 14657; 1:500); anti-BRCA1 (Thermo Fisher Scientific, cat. no. MA1–23164; 1:500); anti-☒H2AX (Sigma Millipore, clone JBW301, cat. no. 05–636; 1:1500); anti-Rad51 (Novus Biologicals, clone 14B4, NB 100–148; 1:300 for IF staining, 1:500 for Western blot); anti-Mre11 (Novus Biologicals, cat. no. NB100–473; 1:1000); anti-☒-tubulin (Sigma Millipore, clone GTU-88, cat. no. T5326; 1:2500); anti-RPA1 (Cell Signaling, cat. no. 2198; 1:1000); anti-pRPAS4/8 (Fortis Life Science, cat. no. A700–009; 1:500); anti-BrdU (BD Biosciences, cat. no. 347580; 1:300); anti-Chk1 (Cell Signaling, cat. no. 2360; 1:1000); anti-pChk1 S345 (Cell Signaling, cat. no. 2348; 1:1000); anti-PARP1 (Abcam, cat. no. ab191217; 1:1000); anti-PCNA (Abcam, cat. no. ab29; 1:3000 for Western blot, 1:1500 for IF staining); anti-histone H3 (Cell Signaling, cat. no. 4499; 1:1000); anti-FLAG (Sigma Millipore, cat. no. F3165; 1:1000 for Co-IP, 1:1500 for Western blot); anti-HA (Sigma Millipore, cat. no. H6908; 1:1000); anti-c-Myc (Cell Signaling, clone D84C12, cat. no. 5605; 1:1000); anti-Cyclin E (Cell Signaling, clone HE12, cat. no. 4129; 1:500); anti-FAAP20 (Novus Biologicals, cat. no. NBP1–93890; 1:1000); anti-FANCG (Novus Biologicals, cat. no. NB100–2566SS; 1:1000); anti-FANCD2 (Abcam, cat. no. ab108928; 1:1000); anti-FANCM (Cell Signaling, clone E5Y9H, cat. no. 38199; 1:500); anti-poly/mono-ADP ribose (Cell Signaling, cat. no. 83732; 1:500); anti-FEN1 (Cell Signaling, clone E5X2T, cat.no. 83104; 1:1500 for Western blot, 1:1000 for IF staining); anti-DNA-RNA hybrid (S9.6) antibody (Kerafast, cat. no. ENH001).

On-target plus human RPA1 siRNA (cat. no. L-015749-01-0005), On-target plus human Rad51 siRNA (cat. no. L-003530-00-0005), and non-targeting control siRNA (cat. no. D-001810-10-05) were purchased from Horizon. PB-TA-ERP2 (cat. no. 80477), pDR-GFP (cat. no. 26475), pCBASceI (cat. no. 26477), and FANCA full-length plasmid (cat.no. 111126) were purchased from Addgene; pDONR^™^221 (cat. no. 12536017) was purchased from Thermo Fisher Scientific; Flag-tagged FEN1 and RNase H1 plasmids (cat. no. OHU15731D and OHu20534D, respectively) were purchased from GenScript.

### Immunoblotting and Immunoprecipitation

For immunoblotting, cells were harvested and lysed with NETN lysis buffer (20mM Tris pH 8.0, 150 mM NaCl, 1 mM EDTA, 1% NP40, 10% glycerol, 1 mM Na_3_SO_4_, and 10 mM NaF) supplemented with protease inhibitors and phosphatase inhibitors (Thermo Fisher Scientific, cat. no. PI78446) followed by 10 minutes of sonication (medium intensity for 30 seconds on/30 seconds off using Diagenode Bioruptor). Protein concentrations were determined by Qubit protein assay kit (Thermo Fisher Scientific, cat. no. Q33212). 30 μg of total protein was loaded in lanes of 4–15% SDS-PAGE gradient gel. Proteins were transferred to the PVDF membrane using BioRad trans-blot turbo transfer system. The membrane was subsequently blocked with 4% milk and then incubated with the indicated antibodies (1:500–1:1000 dilution with TBST) at 4°C with gentle shaking overnight. After washing, the membrane was incubated with a secondary HRP-conjugated goat anti-rabbit antibody (1:5000 dilution in TBST, Cell Signaling, cat no.7074) or goat anti-mouse antibody (1:5000 dilution in TBST, Cell Signaling, cat no.7076) for 1 hour at room temperature, followed by four washes in TBST. Immunodetection was performed by enhanced chemiluminescence detection (Bio-Rad, cat. no. 1705061).

For immunoprecipitation, cells were harvested and lysed in NETN lysis buffer followed by 10 minutes of sonication. The clarified extract was precleared with protein G beads (Cytiva, cat. no. 17061801) for 1 hour at 4° C. 3 μg of appropriate primary antibody was added with protein G beads to the precleared supernatant and incubated overnight at 4° C. Immunoprecipitates were washed four times in NETN buffer and boiled in 1X laemmli sample buffer for 5 minutes. The resulting supernatants were then resolved on SDS-PAGE gel followed by western blotting.

### Immunofluorescent Staining

Cells were cultured on glass coverslips (22×22mm, No.1.5, VWR, cat. no. 16004–302) and treated with the indicated conditions before immunostaining. The cells were fixed with 4% formaldehyde for 15 minutes, permeabilized with 0.3% Triton X-100 for 3 minutes, and blocked with 10% goat serum overnight. The cells were then incubated with primary antibodies (1:100–1:500 dilution in 1% goat serum) for 2 hours at room temperature, followed by washing with PBS and incubation with the appropriate secondary antibodies (Alexa Fluor 594 goat anti-rabbit, cat. no. A11037; Alexa Fluor 594 goat anti-mouse, cat. no. A11005; Alexa Fluor 488 goat anti-rabbit, cat. no. A11034; or Alexa Fluor 488 goat anti-mouse, cat.no. A11001). All were purchased from Thermo Fisher Scientific, diluted at 1:500 in 1% goat serum, and incubated for 1 hour at room temperature in the dark followed by washing. After staining with DAPI, coverslips were mounted onto slides using ProLong gold antifade mounting medium (Thermo Fisher Scientific, cat. no. P36934). Cells were visualized by fluorescence microscopy (Olympus BX-61). Image J software was used to count foci or quantify fluorescence intensity.

For ssDNA foci detection, 0.4 × 10^6^ cells were seeded per well onto a coverslip in a 6-well plate overnight. BrdU (10 μM) was added to the cell culture and kept on for 24 hours. Olaparib (10 μM) and EdU (10 μM) were added to the culture for the last 4 hours and 20 minutes respectively in the course of 24-hour BrdU treatment before cell fixation. Cells were then washed and pre-extracted using CSK buffer (25 mM HEPES pH 7.4, 150 mM NaCl, 0.3 M sucrose, 3 mM MgCl_2_, 1 mM EDTA, 0.5% Triton X-100) for 5 minutes on ice followed by 10 minutes of 4% formaldehyde fixation at room temperature. Cells were blocked using 5% BSA in PBS, and EdU labeling was then performed using Click-iT EdU Alexa Fluor 488 Imaging Kit (Thermo Fisher Scientific, cat. no. C10337) according to the manufacturer’s instructions. Cells were incubated with anti-BrdU antibody (1:300, BD Bioscience, cat. no. 347580) for 1 hour, washed, and incubated with Alex Fluor 488 conjugated secondary antibody for 1 hour in the dark. DAPI staining, mounting onto slides, and fluorescence microscopy imaging and analysis were done as described above.

### Colony Formation Assay

Cells were plated at a density of 1000 cells per 10 cm dish and treated with the indicated conditions. After treatment, cells were grown for 12–14 days at 37°C. 0.5% crystal violet/20% methanol was used to stain cell colonies, and only colonies containing 50 or more cells were counted. Survival rate (%) was calculated by dividing the average number of colonies on treated dishes by the average number on untreated dishes.

### CellTiter-Glo Viability Assay

Cells were seeded on 96-well white clear-bottom plates (1000 cells/well). Compounds were added from DMSO stock solutions on Day 1 using a Tecan D300E dispenser. Cells were grown in the continuous presence of compounds. Cell viability was measured using the CellTiter-Glo 2.0 Cell Viability Assay kit (Promega, cat. no. G9243) according to the manufacturer’s protocol. On Day 8, luminescence was read using a SpectraMax MiniMax 300 imaging cytometer. Percent cell viability was calculated by averaging luminescence among replicates and normalizing to untreated cells.

### Breast Cancer Organoid Experiments

To generate WHIM2 breast cancer organoids, tissue samples were processed as previously described^74^. Briefly, tissues were finely minced and processed for viable cell isolation. Minced tissues underwent enzymatic digestion using collagenase-hyaluronidase mix (StemCell technologies, cat. no. 07912) for 1–2 hours at 37°C, followed by the removal of red blood cells with ammonium chloride solution. The resulting cell clusters were resuspended in growth factor-reduced matrigel (Corning, cat. no. 354230) and plated as 20 μL drops in 24-well plates. BC organoid medium was added to each well and refreshed every 3–4 days. The BC organoid medium consisted of Advanced DMEM:F12 (Gibco, cat. no. 12634010) supplemented with 1X Glutamax, 10 mM Hepes, 1X Penicillin/Streptomycin, 50 μg/mL Primocin, 1X B27 supplement, 5 mM Nicotinamide, 1.25 mM N-Acetylcystein, 250 ng/mL R-spondin 3, 5 nM Heregulin β-1, 100 ng/mL Noggin, 20 ng/mL FGF-10, 5 ng/mL FGF-7, 5 ng/mL EGF, 500 nM A83–01, and 500 nM SB202190. Additionally, 5 μM Y-27632 was included in the medium for the initial three days of culture.

Organoids were passaged upon reaching confluency, typically once a week. For passaging, matrigel was collected from the wells, manually dissociated, and then cells were treated with TrypLE solution (Gibco, cat. no. 12604013) for 8 minutes at 37°C. After neutralizing the enzyme and washing, cells were resuspended in matrigel and reseeded to facilitate new organoid formation. CRISPR-Cas9 editing of WHIM2 organoids was performed using the Neon Electroporator system. Organoids were dissociated into single cells, and 500,000 cells were resuspended in electroporation buffer R. The ribonucleoprotein (RNP) complexes were preassembled using 5 μg/ml recombinant Cas9 2.1 (Thermo Fisher Scientific, cat. no. A36496), 150 pmol sgRNAs targeting FANCA or a non-targeting sgRNA described in the above method, and 1 μg/ml GFP plasmid containing a puromycin resistant marker. Following electroporation, the cells were immediately resuspended in growth factor-reduced matrigel and cultured in organoid media supplemented with Y-27632 to enhance post-electroporation recovery. After 5 days, cells were treated with puromycin for 3 days. Successful editing of the FANCA gene was confirmed by western blot analysis.

For drug treatments, organoids were dissociated, and 5000 cells were resuspended in 10 μL of matrigel, then plated into a 96-well plate. Cells were treated with varying concentrations of Olaparib and DMSO, with three replicates per treatment condition. After 3, 5, and 7 days of treatment, the culture medium was removed, and 100 μL of a 1:1 mixture of CellTiter-Glo 3D Reagent (Promega, cat. no. G9681) and culture medium was added to each well to assess ATP levels as an indicator of cell viability, following the manufacturer’s protocol. Luminescence was measured using a microplate reader (BioTek, Cytation 5 reader).

### Alkaline Comet Assay and BrdU Comet Assay

The alkaline comet assay was performed according to the manufacturer’s instructions (Bio-Techne, cat. no. 4250–050-K). Briefly, cells were untreated or treated with the indicated conditions. Cells were harvested at the recovery time points for single-cell electrophoresis, followed by staining of cell nuclei with SYBR green. The cell comets were visualized by fluorescence microscopy. Comet tail moment was analyzed using OpenComet software.

To specifically detect DNA breaks in nascent strands, cells were untreated or treated with 10 μM Olaparib for 2 hours and then pulse-labeled with 100 μM BrdU for 30 minutes as indicated. Cells were washed and added with fresh culture medium as a chase period for 90 minutes (to measure breaks in nascent strands during OFM). Single-cell electrophoresis was performed as in the alkaline comet assay. After the neutralization step, slides were washed and incubated with mouse primary anti-BrdU antibody (BD Bioscience, cat. no. 347580) overnight at 4 °C in a humid chamber. Following removal of excess primary antibody, slides were then incubated with the mixed two secondary antibodies: goat anti-mouse Alexa Fluor 488 (Thermo Fisher Scientific, cat. no. A11001) and donkey anti-goat Alexa Fluor 488 (Thermo Fisher Scientific, cat. no. A11055) for 1 hour at room temperature to amplify the signal (both diluted 1:250 in PBS/0.1%Tween 20/3% BSA). After washing slides in PBS, the comet images were captured by fluorescence microscopy, and the BrdU comet tail moment was analyzed by OpenComet software.

### Cellular Fractionation

Soluble and insoluble fractions were prepared as previously described^29^. Briefly, cells were collected and lysed for 20 minutes on ice in 200 μl of CSK buffer containing protease inhibitors and phosphatase inhibitors. Soluble and insoluble (chromatin-bound) proteins were separated by centrifugation (10 minutes, 20,000g at 4 °C). Soluble fractions (supernatants) were collected and dissolved in Laemmli sample buffer. Insoluble/chromatin fractions (Pellets) were washed twice in CSK buffer, sonicated for 5 minutes, and subsequently dissolved in Laemmli sample buffer. Western blots were performed as indicated.

### Flow Cytometry Analysis

To determine cell cycle distribution, flow cytometry was performed by propidium iodide staining. Cells were digested with trypsin, washed twice with ice-cold PBS, and fixed with 70% ethanol in a dropwise manner overnight at 4°C. After washing twice with PBS, cells were incubated with 5 μg/ml propidium iodide and 50 μg/ml RNase A in PBS for 1h at room temperature and analyzed by Attune NxT2 flow cytometer. Data were analyzed using Flow Jo software to determine the cell percentage of each cell cycle phase.

### HR Repair Assay

The fluorescence-based HR repair assay was performed as previously described^44^. To determine the effect of FANCA on HR repair, MDA-MB-231 FANCA-knockout clones, MDA-MB-231 Mre11-knockout cells, MDA-MB-231 sgControl cells, and MDA-MB-231 wild-type cells with the integrated HR reporter DR-GFP were generated as previously described. The stable MDA-MB-231 wild-type pDR-GFP cells were transfected with Rad51 siRNA for 2 days. The pDR-GFP cells were further transfected with either an I-SceI-expressing vector or a control vector. EGFP construct was transfected into MDA-MB-231 cells to ensure similar transfection efficiency. Forty-eight hours later, GFP-positive cells were analyzed using a flow cytometer (Attune NxT2, Thermo Fisher Scientific) and Flow Jo software.

### Xenograft Tumor Models

Mouse tumor xenograft experiments were carried out as previously described with some modifications^75^. One million MDA-MB-231 sgControl or sgFANCA cells were injected bilaterally at the mammary fat pad in NOD/Rag mice. Mice bearing tumors of around 5 mm in longest dimension were divided into two groups for each cell line: vehicle group and Olaparib group (50mg/kg with 10% DMSO + 10% 2-hydroxy-propyl-β-cyclodextrin in PBS daily by oral gavage). Tumor volume was subsequently measured every other day using calipers. Mice were sacrificed for tumor dissection on day 28 after the start of Olaparib treatment.

### Immunohistochemistry (IHC) staining

IHC staining was performed on paraffin-embedded tumor tissues that were sectioned onto positively charged slides. Tissue sections were labeled as single IHC stains for antigens using anti-☒- H2AX antibody (Cell Signaling, cat. no. 9718). Slides were dewaxed in bond dewax solution (Leica Biosystems, cat. no. AR9222) and hydrated in bond wash solution (Leica Biosystems, cat. no. AR9590). Heat-induced antigen retrieval was performed at 100 °C in bond-epitope retrieval solution 1 (Leica Biosystems, cat. no. AR9961). After pretreatment, tissues were blocked, and primary antibody was incubated for 1h with anti-☒-H2AX antibody at 1:2000 followed by Novolink Polymer (Leica Biosystems, cat. no. RE7260-K) secondary. Antibody detection with 3,3′-diaminobenzidine (DAB) and hematoxylin counterstain was performed using the BOND Intense R Detection System (Leica Biosystems, cat. no. DS9263). Stained slides were dehydrated and coverslipped with cytoseal 60 (Fisher Scientific, cat. no. 23–244256). A positive control and negative control (no primary control) slide was included for this assay. The stained slides were digitally imaged in the Aperio AT2 (Leica) using a 20x objective. Qupath version 0.5.0 was used for the quantification of gH2AX-positive cells^76^.

### Cloning

To make Tet-On inducible expression vector expressing c-Myc or Cyclin E, an inducible PiggyBac vector PB-TA-ERP2 purchased from Addgene was used as a transpose designation vector in the Gateway cloning protocol (Thermo Fisher Scientific, cat. no 11789020 and 11791020). Briefly, BP reaction was performed by mixing attB-PCR product containing c-Myc or Cyclin E cDNA sequence, pDONR 221, and BP Clonase II enzyme for one-hour incubation at room temperature followed by transformation into Stbl3 competent cells (Thermo Fisher Scientific, cat. no. C737303) to get the correct entry clones confirmed by sequencing. Next, LR reaction was conducted by incubating the entry clone vector, destination vector (PB-TA-ERP2), and LR Clonase II enzyme for one hour at room temperature followed by transformation into Stbl3 competent cells to get the correct destination clones confirmed by sequencing. The doxycycline-induced expression of c-Myc and Cyclin E was confirmed by western blot.

To make FANCA full-length and truncated mutant clones, the FANCA full-length entry clone was made by Gateway BP reaction, and then FANCA truncated mutants (ΔD1, ΔD2, ΔD3, ΔD4, and ΔD5) were made using the Gibson Assembly method based on the FANCA full-length entry clone. All entry clones were confirmed by sequencing. The LR reaction was then performed to make the destination vectors containing the full-length, ΔD1, ΔD2, ΔD3, ΔD4, or ΔD5 mutant using the PB-TA-ERP2 vector. All destination clones were confirmed by sequencing. The expression of FANCA full-length and ΔD1-ΔD5 mutants was confirmed by western blot.

### *In vivo* DDR-CRISPR/Cas9 Screening

A total of 40 virgin female transgenic mice of R26cas9/cas9, Myc OE, and Trp53fl/fllines aged 6–10 weeks were intraductally injected with 10 μl lentivirus at 5 × 10^5^ TU into the fourth mammary glands bilaterally. The lentivirus was generated from our pooled Lenti-CRISPR-Cre-V2-sgRNA DDR plasmid library containing 3908 sgRNAs targeting 309 murine DDR genes with an average of 10 sgRNAs per gene, as well as 834 non-targeting sgRNA controls. Mice were palpated twice a week for tumor detection. 39 tumors were harvested four to five months following intraductal injection for the creation of mammary tumor cell lines. Genomic DNA was extracted from these tumor lines and the CRISPR libraries were prepared and sequenced for confirmation of sgRNAs. Two million mixed cells of these 39 tumor lines were injected into NOD/Rag mice via the fourth mammary glands bilaterally. When the tumor size reached ~ 5mm, mice carrying tumors were divided into different groups: vehicle group, Olaparib group, ATR inhibitor (AZD6738) group, IR group (2 Gy × 4), and IR group (8 Gy × 1), with three mice in each group. Olaparib was dissolved in 10% DMSO + 10% 2-hydroxy-propyl-β-cyclodextrin in PBS and 50mg/kg of Olaparib was applied to each mouse via oral gavage. ATR inhibitor was dissolved in 10% DMSO + 40% propylene glycol + 50% water and 50mg/kg of ATR inhibitor was applied to each mouse via oral gavage. Mouse radiation was performed using an X-Rad smart irradiator at the UNC animal core facility. On day 12 after starting treatment, all mice were sacrificed, tumors were harvested, and genomic DNA was extracted from one million tumor cells using QIAamp DNA Blood Kit (Qiagen, cat. no. 51104) for the next library preparation.

To amplify the lentiCRISPR v2 sgRNA cassette, two rounds of PCR were performed as previously described^77^. Briefly, first-round PCRs were carried out using NEB Fusion DNA polymerase and DDR_CRISPR_Ion_1st_FWD and DDR_CRISPR_Ion_1st_REV with 30 thermal cycles. Second-round PCRs were performed to attach Ion adaptors and to barcode samples using 25 ng of purified product from the first PCR with 10 thermal cycles. All samples’ libraries were then pooled and sequenced on an Illumina iSeq 100 using the 530v1 chip. The sgRNA average abundance was determined from the treated sample relative to the vehicle sample. The volcano plot was made using average abundance and negative log p-value from the t-test for each group.

### *In vitro* Mini CRISPR library generation, lentivirus generation, and Screening

Pooled library cloning. DNA oligonucleotide containing the sgRNA target sequence was synthesized by Eton Biosciences (Supplementary information). A pool of oligonucleotides for making the mini CRISPR library was then amplified by PCR with forward primer ArrayF (TAACTTGAAAGTATTTCGATTTCTTGGCTTTATATATCTTGTGGAAAGGACGAAACACCG) and reverse primer ArrayR (ACTTTTTCAAGTTGATAACGGACTAGCCTTATTTTAACTTGCTATTTCTAGCTCTAAAAC) to produce a suitable double-strand product followed by PCR product purification. The lentiCRISPR v2 vector was digested with BsmBI followed by gel extraction and purification with a Gel purification kit (Qiagen, cat. no. 28704). Gibson assembly was employed to clone the mini library cassette into lentiCRISPR v2 followed by transformation into Endura ElectroCompetent cells (Biosearch Tech. cat. no. 60242–1), according to the manufacturer’s protocol. Four parallel transformations were performed using the same Gibson reaction product and plated into eight 10 cm LB plates containing 100 μg/ml carbenicillin. Colonies were scraped off plates and combined for DNA extraction (Qiagen, cat. no. 51104) followed by library preparation and next-generation sequencing confirmation.

Lentivirus generation. Lentiviruses were generated by transfection of 2 μg pMD2.G, 3 μg psPAX2, and 4 μg mini CRISPR pooled lentiviral library into 293T cells in a 10 cm dish followed by collection of culture medium supernatant at 48 hours and 72 hours after transfection. The supernatant was pooled and then filtered through a 0.45 μm filter. The virus was then concentrated by pelleting at 113,000 × g in a SW28 ultracentrifuge rotor for two hours at 4 °C. The concentrated pellet was dissolved in PBS, aliquoted, and frozen at −80 °C.

Library screening. Our mini DDR-CRISPR pooled lentiviral plasmid library containing 31 sgRNAs targeting corresponding DDR candidate genes selected from the *in vivo* CRISPR screening, as well as 5 non-targeting sgRNA controls, was used to produce lentivirus for infection of cells at a MOI of ~0.3. The lentivirus was added to the cell culture for 24 hours followed by 48 hours of puromycin (2 μg/ml) selection. Once cells grew up, the cells were split, and one million cells were reseeded into a T175 flask in media containing 2 μg/ml puromycin each time. After 8 population doublings, cells were harvested, and genomic DNA was extracted. To amplify the lentiCRISPR v2 sgRNA cassette, two rounds of PCR were performed according to the previously described methods. All sample PCR products were purified after each amplification, and libraries were pooled and sequenced on an Illumina iSeq 100 using the 530v1 chip.

## Supplementary Files

This is a list of supplementary files associated with this preprint. Click to download.

- ExtendeddataFigureslegends.docx
- ExtendedDataFigure1.png
- ExtendedDataFigure2.png
- ExtendedDataFigure3.png
- ExtendedDataFigure4.png
- ExtendedDataFigure5.png
- ExtendedDataFigure6.png
- ExtendedDataFigure7.png
- ExtendedDataFigure8.png
- ExtendedDataFigure9.png
